# Tropism and infectivity of duck-derived egg drop syndrome virus in chickens

**DOI:** 10.1371/journal.pone.0177236

**Published:** 2017-05-08

**Authors:** Min Kang, Se-Yeoun Cha, Hyung-Kwan Jang

**Affiliations:** Department of Veterinary Infectious Diseases and Avian Diseases, College of Veterinary Medicine and Center for Poultry Diseases Control, Chonbuk National University, Iksan, South Korea; Sun Yat-Sen University, CHINA

## Abstract

Egg drop syndrome virus (EDSV) can markedly decrease egg production in laying hens. Duck is the natural host of EDSV. EDSV derived from ducks abrogate egg drop in laying hens. We have previously confirmed that duck-derived EDSVs have a variety of replication activities in chick embryo liver (CEL) cells. However, it is currently unclear whether duck-derived EDSV could display tropism and adaptation in laying hens. This study assessed whether duck-derived EDSV can adapt to laying hens, and estimated the inducing factors. Complete genome sequences of duck-derived EDSVs (D11-JW-012, D11-JW-017, and D11-JW-032 isolates) with various replication efficiency in CEL cells and C10-GY-001 isolate causing disease in laying hens were analyzed to find their differences. Phylogenetic analysis of complete genome sequence revealed that C10-GY-001, D11-JW-032, and strain 127 virus as vaccine were clustered into the same group, with D11-JW-012 and D11-JW-017 clustered in another group. Comparison between D11-JW-012 isolate that poorly replicated and D11-JW-017 isolate that replicated well in CEL cells in same cluster revealed six amino acid differences on IVa2, DNA polymerase, endopeptidase, and DNA-binding protein. These amino acids might be key candidates enhancing cellular tropism in chicken. When the pathogenicities of these isolates in laying hens were compared, D11-JW-032 showed severe signs similar to 127 virus, D11-JW-017 showed intermediate signs, while D11-JW-012 showed almost no sign. Eleven amino acids differed between D11-JW-032 and D11-JW-017, and 17 amino acids were different between D11-JW-032 and D11-JW-012. These results suggest that EDSVs derived from ducks have various pathogenicities in laying hens. Key amino acid candidates might have altered their affinity to tropism of laying hens, causing difference pathogenicities.

## Introduction

Egg drop syndrome virus (EDSV) can infect chickens, causing major economic losses due to its direct effect on egg production and eggshell quality. EDSV outbreak in laying chickens was first reported in 1976 [1]. EDSV is an avian adenovirus belonging to genus *Atadenovirus*. It has a double-stranded DNA genome of 33.2 kb. EDSV is classified as a duck adenovirus serotype 1 [2]. Only one serotype of EDSV has been recognized [3]. EDSV in chicken is thought to have originated from ducks [4]. In ducks, EDSV infections are no clinical sign. However, EDSV antibodies have repeatedly been found in various domesticated ducks and geese worldwide. Global serologic evidence of EDSV infection in a variety of wild waterfowl species has been reported [5–8]. Although EDSV might have been introduced to chickens by contaminated vaccine [1], the epizootiological significance of EDSV infection in ducks is unclear.

Although EDSV isolates from ducks and chickens are serologically similar, EDSVs isolated from ducks fails to infect chickens or reduce egg production in laying hens [9]. Experimental contact transmission of EDSV from ducks to chickens, and pathogenicity increases through multiple passages in chickens have been reported [10]. On other hand, EDSV isolated from healthy duck flock resulted in EDS signs in chickens similar to equal decrease in egg production produced by the 127 virus [11]. EDSVs isolated from ducks with high, intermediate, or low replication without prior chicken cell adaptation have been identified using chick embryo liver (CEL) cells. EDSV isolates D11-JW-017 obtained from Pekin ducks have high replication ability in CEL cells [12]. Their replication ability is similar to the C10-GY-001 outbreak strain isolated from laying hens. However, the D11-JW-012 isolate poorly replicates in CEL cells [12]. Various pathogenicities of EDSVs derived from ducks in laying hens have been reported [9–11]. Genetic determinants of host tropism and virulence are currently unknown. Information of genome and pathogenic mechanisms unique to EDSV is limited and genome sequence is available for only one isolate in GenBank.

The apparent relationship between EDSV biodiversity and their hosts infers that host adaptation might be the driving force of EDSV diversification and evolution. However, this hypothesis has not been supported by appropriate studies designed to dissect the evolutionary mechanisms in EDSV. To improve our understanding of genetic diversity at genome level for EDSVs that reside in distinct hosts and gain insights into their evolutionary path, we performed comparative genomic and pathogenic analyses of EDSVs isolated from chickens and ducks in this study.

## Materials and methods

### Ethics statement

All experimental work involving animals was approved (CBU 2012–0048) by The Chonbuk National University Animal Ethics Committee in accordance with the guidelines of The Korean Council on Animal Care.

### Virus

This study used ESDVs isolated from chickens and ducks from South Korea (Table 1). In addition, EDS 127 virus [1, 13] was used. These isolates were inoculated into the allantoic cavity of 10-day-old EDSV-free embryonated duck eggs obtained from a local source. A random analysis of yolk samples indicated that these eggs were free of hemagglutinin inhibition antibodies to EDSV. Live embryos were chilled 5 days after inoculation. The allantoic fluid was harvested and used as viral source after determining HA titer. A second passage was carried out for field isolates. All virus stocks were stored at −80°C until use.

**Table 1:** Complete genome sequence analysis compared to that of the strain 127 virus and the characteristics of egg drop syndrome virus (EDSV) field strains used in this study.^a^

Strain	G+C content (%)	Number of SNPs	Similarity (%)	Accession No.^b^	Country	Origins
127	43.01			Y09598	Netherlands	Laying birds
C10-GY-001	43.01	4	99.99	KJ452173	South Korea	Laying chicken
D11-JW-012	42.99	78	99.76	KJ452170	South Korea	Pekin Ducks
D11-JW-017	43.00	68	99.81	KJ452171	South Korea	Pekin Ducks
D11-JW-032	43.01	4	99.99	KJ452172	South Korea	Pekin Ducks
FJ12025	43.03	50	99.90	KF286430	China	Muscovy Ducks

^a^Numbers of single nucleotide polymorphisms (SNPs) and similarities compared to the strain 127 virus.

^b^Complete genome sequence data are available at GenBank.

### Viral DNA isolation

Phenol-chloroform method was used to extract viral DNA. Briefly, proteinase K (10 mg/ml) was added to viral suspensions and incubated at 37°C overnight. Phenol:chloroform:isoamyl alcohol (25:24:1, v: v: v) was then added to the solution and mixed well, followed by centrifugation at 12,000 × g for 10 min at 4°C. Following another chloroform treatment, the aqueous phase was precipitated with chilled ethanol through centrifugation (12,000 × g for 10 min at 4°C). The pellet was washed with 70% ethanol and dried. DNA was dissolved in nuclease-free water.

### Sequencing

Selected isolates were sequenced using the Personal Genome Machine (PGM; Life Technologies, Darmstadt, Germany). This kit includes Ion Shear Plus Reagents for enzymatic fragmentation of genomic DNA and Ion Plus Fragment Library Kit to prepare sequence library using enzymatically fragmented DNA. A total of 1 μg of genomic DNA was used to construct the library. Template-positive Ion Sphere Particles (ISPs) containing clonally amplified DNA were produced using the Ion OneTouch 200 Template Kit v2 (for 200 base-read libraries) on the Ion OneTouch Instrument. The instrument was also used to enrich ISPs intended for the Ion PGM System using Ion PGM 200 Sequencing Kit and Ion 316 sequencing chip. A total of 105 sequencing cycles resulted in a mean read length of 200 nucleotides. Single nucleotide polymorphisms (SNPs) and indels of MC58 genome sequence were extracted with the newest version of CLC Genomes Workbench software (CLCbio, Aarhus, Denmark).

### Phylogenetic analysis

Complete genomes of Korean EDSV isolates were compared to sequences of selected vaccine strain 127 virus and FJ12025 isolated from Muscovy ducks. Complete genome nucleotide sequences were obtained from GenBank. Their deduced amino acid sequences were aligned using Mega ver. 4.0 software packages (DNAStar, Madison, WI, USA). Phylogenetic tree was prepared using neighbor-joining method and bootstrap testing.

### Genome assembly and analysis

DNA sequences were assembled using Seqman program (DNAStar) and mapped manually. Complementary genome sequences of EDSV field isolates were compared to the sequences of selected EDSV from various animals and geographic locations. Complete genome nucleotide sequences were obtained from GenBank. Their deduced amino acid sequences were aligned using Mega ver. 4.0 software. Open reading frames (ORFs) were predicted using the ORF finder function of Geneious software package.

### Growth of isolates in avian cells

To compare the growth efficiency of chicken primary embryo liver cells (CEL) and duck primary embryo liver cells (DEL), cells were prepared from 14-day-incubated chickens or 17-day-incubated duck embryos, respectively. Cells were grown in Dulbecco’s modified Eagle’s medium (Gibco, Grand Island, NY, USA) supplemented with 5% fetal calf serum and antibiotics at 37°C in an atmosphere of 5% CO_2_. Each of two-fold virus dilutions from 256 HAU/25 μl was added to plate and incubated for 1 h. After removing the inoculate, fresh medium was added to each well, and the cells were cultured for 14 days. We observed daily for cytopathic effects (CPE) and calculation of virus titre using the Spearman-Karber method.

### Experimental infection in chicken

Five groups of 25 30-week-old specific pathogen-free, white Leghorn laying chickens was used. The chickens were infected orally with 0.5 ml duck embryo allantoic fluid containing a virus quantity of 10^6^ median embryo infective doses (EID_50_) with phosphate buffered saline (PBS), 127 virus, D11-JW-012, D11-JW-017, D11-JW-032 isolates, respectively, and observed for a further 5 weeks. Clinical observations and egg production were recorded and egg quality was assessed daily. To provide serological evidence of infectivity of virus used for challenge, blood samples and cloacal swabs were collected from infected laying chickens from 1 week to termination of experiments (5 weeks after infection). All chickens were fed in isolation (Three-Shine, Daejeon, Korea).

### Identification of virus by polymerase chain reaction

Polymerase chain reaction (PCR) was carried out with a set of primers specific for the interior hexon gene to confirm EDSV: EDS H5 (5´-TTCTGTCACCGATAAAGGT-3´) and EDS H6 (5´-AGTTATTCCAAATGGGCAT-3´) [14]. Amplification was performed by pre-denaturation at 94°C for 2 min, 30 cycles of denaturation at 94°C for 1 min, annealing at 62°C for 1 min, extension at 72°C for 2 min, followed by a final extension step of 2 min at 72°C. Samples were obtained from cloacal swabs every week.

### Hemagglutination inhibition (HI) test

Serial two-fold dilutions of test sera were made in 25 μl volumes in wells of a 96-well V-bottomed plate using PBS pH 7.2 as the diluent. Then, 25 μl of EDS antigen (127 strain) containing four hemagglutinating units was added to each well. The constituents were well mixed, and the plate was incubated for 15 min at room temperature. An equal volume of 1% chicken red blood cells was added to each well and incubated for 40 min at room temperature. The test was assessed by tilting the plate.

### Statistical analysis

All data were analyzed using one-way analysis of variance and SPSS 11.0 software (SPSS Inc., Chicago, IL, USA). Significant differences among the groups were further analyzed using the Tukey-Kramer method. A *P*-value < 0.05 was considered significant.

## Results

### Genome organization

Genome sequences of Korean isolates of EDSV derived from laying chickens (C10-GY-001) and Pekin ducks (D11-JW-012, D11-JW-017, and D11-JW-032) were fully sequenced, assembled, and submitted to NCBI GenBank database. The genome sizes of these Korean EDSVs were identical at 33,213 base pairs. Their genome sizes were also identical to those of 127 and FJ12025 viruses. The nucleotide sequences of these complete genomes were analyzed. These Korean isolates of EDSV shared 99.76–99.9% sequence similarities with published sequences of 127 and FJ12025 viruses isolated from chickens and Muscovy ducks, respectively. Their G + C contents were 42.99–43.01%. A total of 4–78 SNPs were identified (Table 1). The nucleotide and amino acid sequences of these strains (four Korean isolates and one Chinese isolate) were compared to those of strain 127 virus retrieved from GenBank. As a result, genetic similaritises were 99.5–100% in nucleotide sequences and 99.5–100% in amino acid sequences.

### Phylogenetic analysis

**Fig 1:**
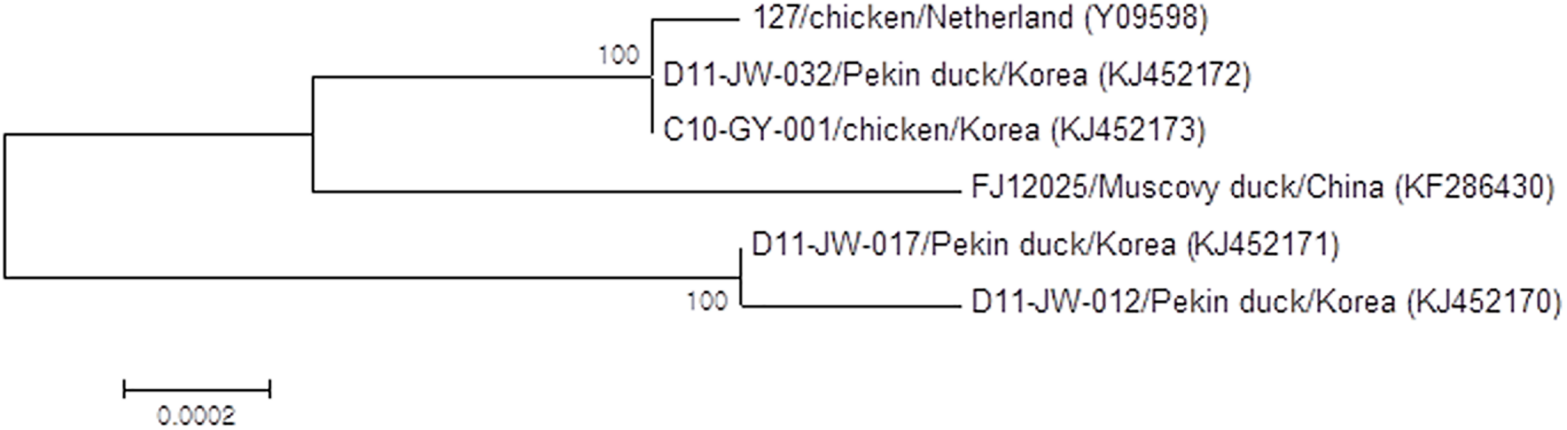
Phylogenetic analysis of complete genome sequences of four Korean egg drop syndrome virus (EDSV) isolates. The phylogenetic tree was prepared using the neighbor-joining method and bootstrap testing. Numbers over the branches indicate the percentage of 1,000 bootstrap replicates supporting the branch.

A phylogenetic tree was constructed to determine the genetic relationships of Korean isolates with other EDSV and adenovirus isolated from other animals or human beings. Phylogenetic analysis of complete genome sequences (Fig 1) revealed that all these isolates could be divided into two groups. Genome sequences of C10-GY-001 and D11-JW-032 were very closely related to those of strain 127 virus. However, their relationship with D11-JW-012 and D11-JW-017 field isolates was more distant.

### Analysis of mutated amino acids with associated cell tropism

Amino acid sequence and mutations were analyzed to identify factors causing difference in propagation ability. Alignment of amino acid sequences of D11-JW-032 with vaccine strain 127 only revealed two mutations on DNA polymerase (Pol) and fiber. However, eleven mutations on large T-antigen (E1B-55 KDa protein), Iva2, Pol, precursor terminal protein (pTP), IIIa, 100K, Fiber, and 34-kDa protein were found between amino acid sequences of D11-JW-032 and D11-JW-017. Alignment of amino acid sequences of D11-JW-032 and D11-JW-012 revealed 17 mutations on large T-antigen (E1B-55 KDa protein), Iva2, Pol, pTP, IIIa, endoproteinase (EP), DNA binding protein (DBP), 100K, fiber, and 34-kDa protein (Table 2). The same HA titer was inoculated using serial 2-fold virus dilutions to visually assess CPEs and calculation of titers (Table 2).

**Table 2:** Amino acid mutations unique to the strain 127 virus.

Region	Protein	Amino acid position	Strains					
			Chicken		Duck			
			127	C10-GY-001	D11-JW- 032	FJ12025	D11-JW- 017	D11-JW -012
E1B	Large T-antigen (55-KDa protein)	94	I	I	I	I	M	M
E2B	IVa2	55	I	I	I	V	V	V
		179	N	N	N	N	N	T
		192	K	K	K	K	K	Q
	Pol	77	L	V	V	V	V	V
		551	V	V	V	V	V	L
		798	N	N	N	N	Y	Y
	pTP	313	T	T	T	P	P	P
L1	IIIa	236	Q	Q	Q	Q	H	H
		304	H	H	H	Q	Q	Q
L3	Hexon	196	G	G	G	A	G	G
	EP	101	D	D	D	D	D	A
		177	S	S	S	L	S	S
E2A	DBP	386	E	E	E	E	E	D
		387	D	D	D	D	D	#
L4	100K	57	F	F	F	L	L	L
		685	A	A	A	V	V	V
L5	Fiber	86	K	R	R	R	R	R
		333	Y	Y	Y	D	D	D
		354	V	V	V	A	V	V
E4	34-kDa protein	193	L	L	L	L	V	V
DEL cells		Log _2_TCID_50_/ml	16.0 ± 0.0.	11.0 ± 0.3	11.0 ± 0.3	-	12.0 ± 0.1	10.9 ± 0.1
CEL cells			15.0 ± 0.2	9.0 ± 0.2	9.0 ± 0.2	-	10.0 ± 0.1	5.0 ± 0.2

# Premature stop codon.

Abbreviations: EP, endoproteinase; DBP, DNA binding protein; pTP, precursor terminal protein; POL, DNA polymerase; DEL, duck embryo liver; CEL, chicken embryo liver.

### Experimental response of the disease and gross lesions

**Fig 2:**
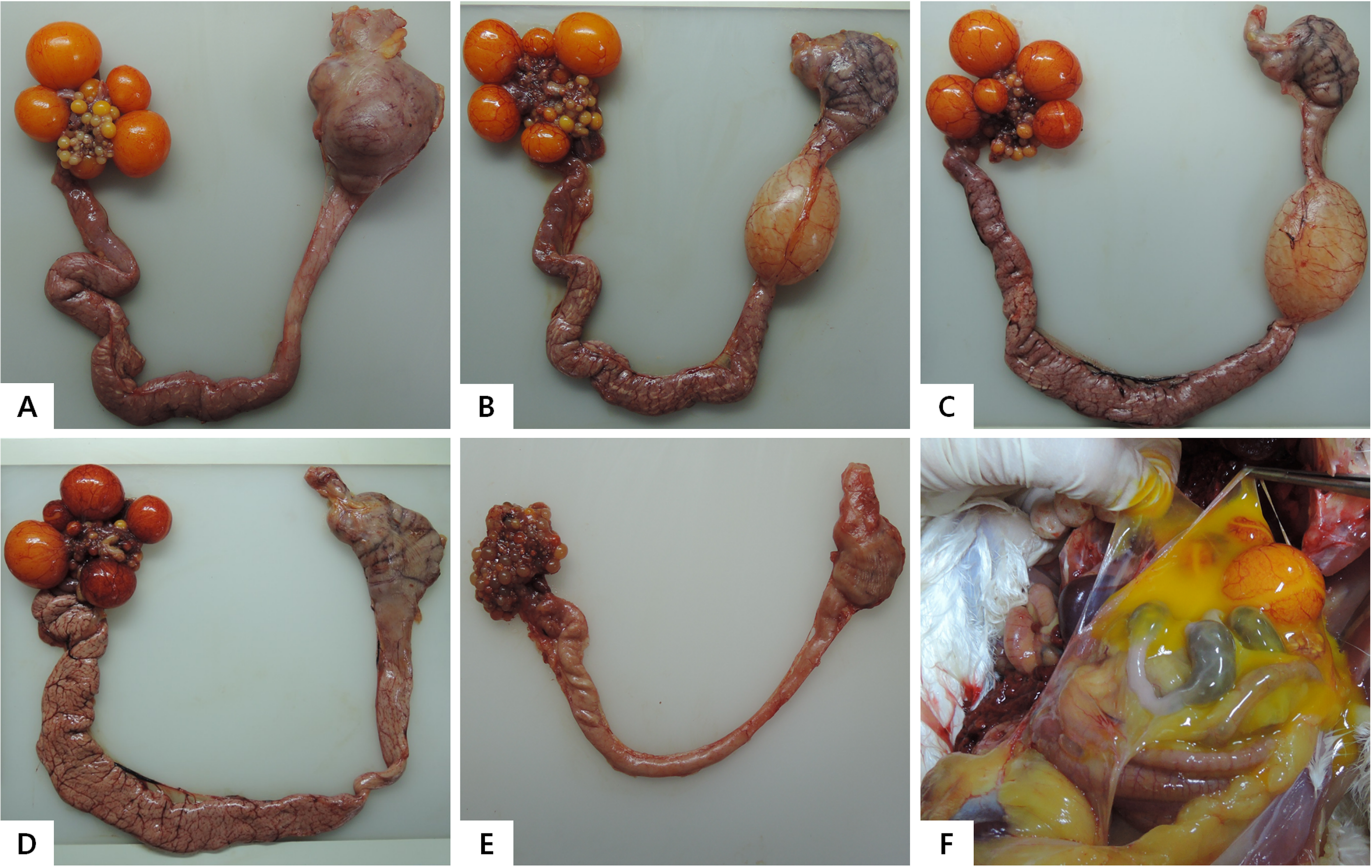
Gross lesions change of the experimentally infected chickens. Reproductive system of ovary and oviduct of chicken inoculated with PBS control (A), D11-JW-012 (B), D11-JW-017 (C), D11-JW-032 (D), or 127 virus (E). Ruptured ovarian follicles and peritonitis were also found in chickens infected with 127 virus (F).

Egg production, external egg quality, egg weights, and eggshell thickness were decreased in white Leghorns laying hens infected with D11-JW-032 isolate like 127 virus. Production decrease with infected D11-JW-032 and 127 virus was first evident 5 or 7 days post inoculation. It was the greatest at 17 to 24 days after inoculation. The decline in external egg quality was first evident at 14 days post inoculation. It was the greatest at 16 or 23 days after inoculation. Egg weight was decreased after 7 days after inoculation and significant difference to PBS and D11-JW-012 (*P*<0.05; Table 3). Cracked, thin-shelled, soft-shelled, and shell-less eggs were characteristic of eggs of infected chickens. Eggs also had ridged or misshapen shells. Egg production and quality returned to PBS control levels at 35 days postinoculation. Egg weights of chickens infected with D11-JW-017 were lower than those of group PBS control. They also showed egg production drop for 3 days. Such egg drop affect was the greatest at 15 days post-inoculation. However, white Leghorns laying hens infected with D11-JW-012 isolate showed no adverse affects on egg production, external egg quality, or eggshell thickness. Serological response results of HI tests are shown in Table 4. Virus infected sera taken 7 days after innoculation showed titers, and titers associated with 127 and D11-JW-032 were higher and earlier than those associated with others (D11-JW-012 and D11-JW-017). Persistence of virus in cecal swabs after inoculation were examined; all virus infected groups could only be detected up to 7 days. At necropsy, severe ovarian hemorrhage, ovaritis, and regression were consistently observed in D11-JW-032 and 127 virus infected laying hens. Ruptured ovarian follicles and peritonitis were also found in chickens infected with 127 virus and atrophy of oviducts. Hemorrhagic follicles on ovaries were found in chickens infected with D11-JW-032. These chickens also displayed decreased egg production. Chickens infected with D11-JW-012 or D11-JW-017 isolate were covered with mild blood vessels and areas of red discoloration in ovarian and edema of oviducts (Fig 2).

**Table 3:** Mean weight of laying chickens infected with EDSVs.

Inoculum	Egg weight (mean ± SD) of weeks of post inoculation					
	0	1	2	3	4	5
PBS	61.6 ± 2.9	62.8 ± 4.0^a^	62.5 ± 1.9 ^a^	62.7 ± 1.9 ^a^	63.5 ± 2.5	64.2 ± 3.6
127	62.5 ± 1.8	52.3 ± 2.1^b^	55.0 ± 1.0 ^b^	54.0 ± 1.0 ^b^	57.7 ± 5.9	61.4 ± 1.4
D11-JW-032	62.1 ± 1.5	52.5 ± 2.6^b^	56.5 ± 2.3 ^bc^	54.7 ± 4.7 ^b^	56.6 ± 4.7	62.6 ± 2.7
D11-JW-017	61.0 ± 1.1	60.1 ± 1.4^a^	60.4 ± 1.5 ^ac^	53.4 ± 2.3 ^a^	62.4 ± 2.2	64.0 ± 0.9
D11-JW-012	62.3 ± 2.4	62.8 ± 2.7^a^	63.7 ± 0.9 ^a^	64.2 ± 1.0 ^a^	64.1 ± 3.1	64.5 ± 3.3

The superscripts ^a, b, c^ denotes a significant difference statistically (One-Way ANOVA, SPSS 11.0).

**Table 4:** Serum antibody titers of laying chickens infected with EDSVs.

Inoculum	Reciprocal of HI titer (log2; mean ± SD) of weeks of post inoculation					
	0	1	2	3	4	5
PBS	0.0 ± 0.0	0.0 ± 0.0	0.0 ± 0.0	0.0 ± 0.0	0.0 ± 0.0	0.0 ± 0.0
127	0.0 ± 0.0	6.3 ± 0.6	7.7 ± 1.5	8.0 ± 0.0	8.0 ± 0.0	7.7 ± 0.6
D11-JW-032	0.0 ± 0.0	6.0 ± 1.0	7.0 ± 1.7	8.3 ± 1.5	8.3 ± 1.5	6.7 ± 1.5
D11-JW-017	0.0 ± 0.0	2.7 ± 0.6	9.0 ±1.0	7.3 ± 0.6	7.3 ± 0.6	7.3 ± 0.6
D11-JW-012	0.0 ± 0.0	2.3 ± 0.6	6.7 ± 1.5	8.0 ± 0.0	7.3 ± 0.0	7.7 ± 0.6

## Discussion

Duck-derived EDSVs displayed various pathogenicities in laying hens. This might be due to key amino acids differences. Four EDSVs from ducks (D11-JW-012, D11-JW-017, D11-JW-032 isolates) or chickens (C10-GY-001) were used in this study. C10-GY-001 isolate was an outbreak strain isolated from chickens. It could infect reproductive organs and induce lower egg production. In our previous study that investigated host adaptation of duck-derived EDSVs to laying hens, cell growth efficiency was assessed using hemagglutinin (HA) titer and cytopathic effects in duck embryo liver and chick embryo liver (CEL) cells [12]. D11-JW-017 and D11-JW-032 isolates propagated well in CEL cells similar to C10-GY-001 isolate, or vaccine strain 127 from chickens, but D11-JW-012 isolate propagated poorly compared to D11-JW-017 isolate. The D11-JW-017 and D11-JW-032 isolates showed results that differed from a previous study in which the HA titer for duck-derived EDSVs was lower than the HA titer for chicken-derived EDSVs [10]. To determine the factor that might affect the replication ability of EDSV in CEL, we compared complete genome sequences and amino acid sequences of D11-JW-012, D11-JW-017, D11-JW-032, G10-GY-001, and vaccine strain 127 virus. We found 100% identities in genome sequences was found between D11-JW-032 and G10-GY-001 isolate. In addition, 99.76–99.9% similarities were found when these isolates were compared to each other. Comparing amino acid sequences between D11-JW-012, which replicates poorly in CEL, and D11-JW-017, which replicates well in CEL, revealed six mutations: T179N and Q192K on IVa2, L551V on DNA polymerase, A101D on endopeptidase, and D386E and ^#^387D on DNA-binding protein (Table 2).

These mutations might have contributed to the adaptation of ESDVs to chicken cells. IVa2 protein is multifunctional. It has been implicated as a transcriptional activator of viral major late promoter and a key component in the packaging of the viral genome [15]. Proteins generated from the E2 region of adenovirus play a major role in viral DNA replication in which early viral transcription can result in expression of three viral genes, including a precursor terminal protein, polymerase, and DNA-binding protein [16, 17]. Endopeptidase is required early in an infection to mediate the release of virus particles from endosomes [18, 19]. These results suggest that factors determining host cell growth efficiency of EDSV are associated with steps in DNA replication, late transcription, or assembly of viron particles, and lysis of host cells.

EDSV causes economic loss to laying chickens. The infection is characterized by drops in egg production accompanied by the production of soft-shelled or shell-less eggs or failure to reach peak production [1, 3]. EDSVs isolated from ducks were reported to have low replication pattern in cells obtained from chickens without clinical disease [9], although contrary findings were reported soon thereafter [11]. We investigated whether isolates from ducks could cause substantial decrease in egg production of laying chickens by experimental infections. Through experimental infections of these isolates with differences in amino acid sequences and replication abilities in CEL cells, the D11-JW-032 isolate was associated with decreases in egg production, external egg quality, egg weights, and eggshell thickness, while D11-JW-017 showed egg production drop for 3 days with decreased egg weight. Isolate D11-JW-012 showed similar results compared to PBS control. These results suggest that isolate D11-JW-012 has typical characters of EDSV isolated from ducks, that the D11-JW-017 isolate is an intermediate form between duck and chicken, and that the D11-JW-032 isolate is an adaptive form in chicken. Comparing amino acid sequences of D11-JW-017 and D11-JW-032 revealed 11 amino acid differences (Table 2), suggesting these amino acid mutations on proteins, such as large T-antigen (E1B-55 KDa protein), Iva2, Pol, pTP, IIIa, 100K, fiber, and 34-kDa protein, might have increased the affinity of EDSV to reproductive organ in laying hens. Protein IIIa as a structural component contacts the outer region of the hexon capsomer; it is cleaved during capsid assembly [20, 21]. Viral 100K protein is a nonstructural protein present in the nucleus and cytoplasm of adenovirus infected cells late in the infection. In the cytoplasm, binding of 100K to the C terminus of translation initiation factor eIF4G can displace MnK1 and lead to inhibition of cap-dependent cellular mRNA translation [22]. In the nucleus, the 100K protein is involved in the folding of hexon polypeptides into trimers. It plays a role in hexon nuclear import [23]. Proteins encoded by the E4 region are involved in several levels of regulation of cellular and viral gene expression, viral DNA replication, late viral assembly, E2 expression, and adeno-associated virus helper function [24–26]. The major host specificity factor in adenovirus is the fiber. The infection starts with the binding of fibers to a specific receptor on the cell surface. The carboxy-terminal portion of the fiber head is referred to as the knob. It can bind to target cells [27, 28]. It is expected that other factors including fiber mutations might also play important roles in reproductive organ specificity of EDSV in chickens.

These results suggest that field EDSV isolates from ducks might have adapted to chickens or acquired high replication efficiency in chickens. It is currntely unclear whether this is common. Our results also reveal that amino acid mutations might be involved in the adaptation of EDSV in chicken. To the best of our knowledge, this is the first report of complete genomes of Korean EDSV isolates with different pathogenicities. The role of these amino acids in EDSV needs to be determined using recombinant viruses.

## Supporting information

S1 ARRIVE Checklist NC3Rs arrive guidelines checklist. (PDF)
